# Clinical Implications of Lymph Node Thyroglobulin in Papillary Thyroid Carcinoma Metastases: Independent from Thyroglobulin Antibody Interference

**DOI:** 10.3390/ijms26115340

**Published:** 2025-06-01

**Authors:** Ping-Chen Kuo, Wen-Chieh Chen, Wei-Che Lin, Shun-Yu Chi, Yi-Hsiang Chiu, Ya-Chen Yang, Chen-Kai Chou

**Affiliations:** 1 Division of Endocrinology and Metabolism, Department of Internal Medicine, Kaohsiung Chang Gung Memorial Hospital, Kaohsiung 833, Taiwan; benson41512@gmail.com (P.-C.K.); chingjing@cgmh.org.tw (W.-C.C.);; 2 Department of Diagnostic Radiology, Kaohsiung Chang Gung Memorial Hospital, Kaohsiung 833, Taiwan; alex@cgmh.org.tw; 3 Departments of Surgery, Kaohsiung Chang Gung Memorial Hospital, Kaohsiung 833, Taiwan; abraban@cgmh.org.tw; 4 College of Medicine, Chang Gung University, Kaohsiung 833, Taiwan

**Keywords:** fine-needle aspiration thyroglobulin (FNA-Tg), washout thyroglobulin, thyroid cancer, cervical lymph node metastasis

## Abstract

Papillary thyroid carcinoma (PTC) frequently involves cervical lymph node (LN) metastases and is a major determinant of prognosis and recurrence. However, cytology alone has limitations. Fine-needle aspiration thyroglobulin (FNA-Tg) has emerged as a promising diagnostic marker, although its cutoff value remains controversial, particularly in patients with thyroglobulin antibodies (TgAbs). We retrospectively analyzed 63 LNs of 60 patients with PTC at a single medical center. Patients underwent FNA-Tg measurements and concurrent cytological evaluation. Diagnostic performance metrics, including sensitivity, specificity, positive and negative predictive value, and overall accuracy, were evaluated; the cutoff value was determined; and the potential influence of factors such as TgAb on FNA-Tg levels was investigated. A cutoff value of 4.23 ng/mL for FNA-Tg achieved 100% sensitivity and 90.2% specificity, with an overall accuracy of 93.6%. TgAb positivity did not significantly affect the diagnostic performance in patients with FNA-Tg. FNA-Tg might be useful for detecting local LN recurrence and providing valuable diagnostic insights, particularly in patients with residual thyroid tissue or positive TgAbs.

## 1. Introduction

Over the past four decades, the incidence of thyroid cancer, which includes papillary, follicular, medullary, and anaplastic thyroid carcinoma, has increased significantly, primarily driven by a rise in papillary thyroid carcinoma (PTC), which accounts for approximately 70–96% of all thyroid cancer cases. This increase may reflect improvements in imaging technologies, leading to more frequent detection of subcentimeter nodules that are subsequently diagnosed as PTC. Nonetheless, despite the typically slow progression and favorable prognosis of PTC, its rising incidence has raised concerns about disease-related mortality [1,2,3,4]. A major factor associated with disease-specific mortality is cervical lymph node (LN) metastasis [5,6], which is also the most common recurrence site during follow-up [7]. Cervical LN recurrence has been observed in approximately 1.4–5.5% of patients despite comprehensive treatment, including total thyroidectomy and radioiodine therapy [8,9]. Therefore, distinguishing LN metastases from benign changes is important to avoid higher disease-specific mortality.

Postoperative follow-up of cervical LNs primarily involves measurement of serum thyroglobulin (Tg) and neck ultrasonography. We evaluated the LNs for malignant features on neck ultrasound, such as microcalcifications, hyperechogenicity, and round shape. However, imaging findings are not always reliable, with accuracies varying between 48% and 90%, depending on specific characteristics [10]. Tissue proof with fine-needle aspiration cytology (FNAC) was used to determine the presence of metastasis. Several studies have reported sensitivities ranging from 66% to 95% and specificities ranging from 40% to 100% for FNAC in detecting malignant LNs [11,12,13]. Certain limitations exist that may be attributed to micrometastases, partial involvement, or cystic changes in LNs, resulting in insufficient samples or even false negatives in cytological analysis [14].

In a pioneering study conducted by Pacini et al. [15], several researchers proposed the use of Tg from fine-needle aspiration washout fluid (FNA-Tg) as a valuable tool for the detection of cervical LN metastases [16,17,18,19,20,21]. There are controversies regarding the cutoff value of FNA-Tg for diagnosing malignant LNs, with values ranging from 0.2 ng/mL to 50 ng/mL [22]. Multiple factors, such as the presence of thyroglobulin antibodies (TgAbs), may influence these values. Moreover, there is currently a lack of extensive local data from Taiwan to establish reliable cutoff values.

Our primary objective was to evaluate the efficacy of FNA-Tg values in detecting neck LN metastases among PTC patients with PTC who had undergone total thyroidectomy or radiofrequency ablation (RFA) by analyzing FNA-Tg levels in 63 patients at a single medical center. We also examined various factors that could potentially influence the FNA-Tg levels. Notably, TgAb is of particular interest, as its presence can substantially compromise the accuracy of serum Tg monitoring during follow-up.

## 2. Results

In this study, 63 FNA-Tg measurements were obtained from 60 patients; approximately 76.2% of these patients were women. The median age at diagnosis was 50 years and the median size of the primary thyroid tumor was 15 mm. At the time of diagnosis, 44.4% of the patients exhibited LN metastases, whereas distant metastases (3.2%) and mortality (0%) were nearly absent. In terms of ultrasonographic features, the absence of hilum was the most frequently observed characteristic, present in 60.3% of cases. This was followed by hyperechoic content (22.2%), cystic change (11.1%), and calcification, which was the least common finding (4.7%). Regarding laboratory data, 14.3% of patients were positive for TgAb. The median serum Tg and serum TSH levels were 1.67 ng/mL and 0.4 µIU/mL, respectively (Table 1).

Of the 63 LNs, 22 were diagnosed as metastatic malignant LNs, while 41 were classified as benign based on FNA cytology. No significant differences were noted between the two groups in terms of basic characteristics such as sex and age. Regarding primary cancer at the time of diagnosis, there was initially a higher proportion of patients with LN and distant metastases in the malignant group. However, further analysis revealed no significant differences between the two groups in terms of distant metastases, primary tumor size, or the presence of LN metastases (N1 stage). Similarly, LN size did not differ significantly between the two groups. On ultrasound imaging, calcification in malignant LNs was significantly associated with malignancy, whereas other features, such as cystic change, hyperechoic content, and absence of hilum, showed no significant difference between the two groups. With regard to laboratory findings, the malignant group had higher FNA-Tg and serum Tg levels. However, there were no significant differences in the TgAb or TSH levels between the groups (Table 1).

**Figure 1:**
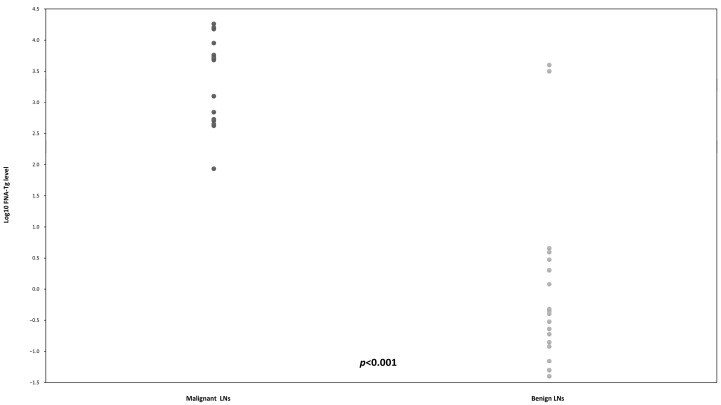
The results of log-transformed FNA-Tg between malignant and benign groups. LN: lymph node; FNA-Tg: thyroglobulin from fine-needle aspiration washout fluid.

FNA-Tg levels in malignant cases ranged from 442 to 18,261 ng/mL, with a median value of 5000 ng/mL and an interquartile range of 4447 ng/mL. The LN sizes ranged from 26 mm to 8 mm, primarily located in levels III (3 cases), IV (7 cases), and VI (11 cases), with only 1 case in level V. The median serum Tg level at the time of comparison was 4.8 ng/mL, with an interquartile range of 20.4 ng/mL. Most values were between 0.5 and 5 ng/mL, with only three cases showing significant increases (22, 42, and 59 ng/mL). On the other hand, in the benign group, the FNA-Tg levels ranged from 0.04 ng/mL to 5000 ng/mL, with a median value of 0.05 ng/mL and an interquartile range of 0.38 ng/mL. The largest LN measured 15 mm, with most measuring less than 10 mm. The median value of serum Tg concentrations at the time of comparison was 0.7 ng/mL, with an interquartile range of 10.8 ng/mL. The FNA-Tg levels in the two groups are presented in Figure 1. The FNA-Tg values were log-transformed to enhance clarity, and a significant difference was observed between the two groups (*p* < 0.001).

**Figure 2:**
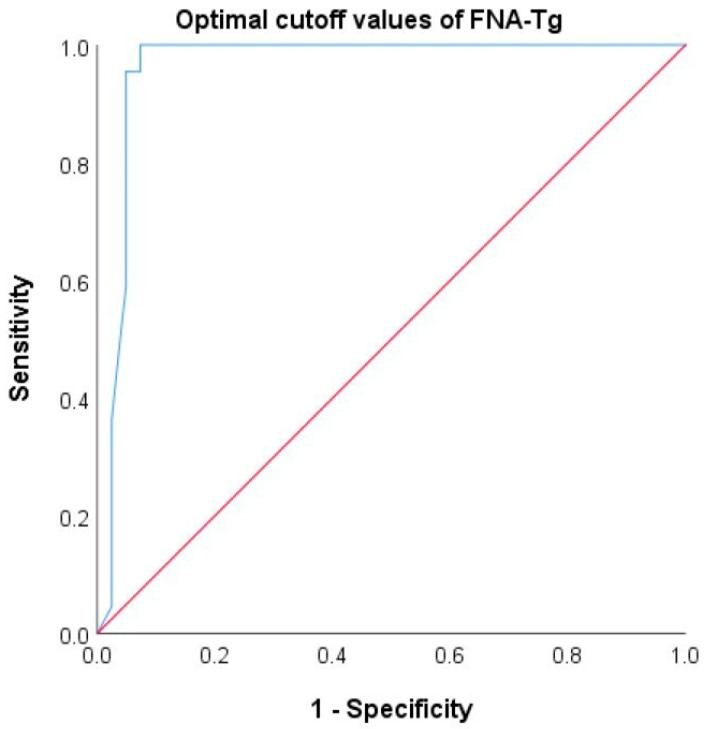
Optimal cutoff values of FNA-Tg by ROC curve. The AUC is 0.962 (95% CI, 0.876–1.00). Blue line: ROC curve, red line: reference line for random chance. FNA-Tg: thyroglobulin from fine-needle aspiration washout fluid.

Using a cutoff Tg level of 4.23 ng/mL to distinguish benign from metastatic LNs resulted in a sensitivity of 100% and a specificity of 90.2%, with an AUC of 0.962 (95% CI, 0.876–1.00) (Figure 2). Table 2 summarizes the diagnostic performance metrics of various criteria, including large-scale studies and international guidelines. Notably, both the American Thyroid Association (ATA) guidelines and Moon et al.’s study from Korea support a cutoff value of FNA-Tg > 1 ng/mL [17,23]. In addition, a study by Jiang et al. from Taiwan proposed that LN-FNA-Tg greater than serum Tg and higher than 1 ng/mL could be used as diagnostic criteria [24]. We compared the sensitivity, specificity, positive predictive value, negative predictive value, and overall accuracy. The accuracies of our findings and those of these criteria were 93.6%, 87.3%, and 92%, respectively.

**Figure 3:**
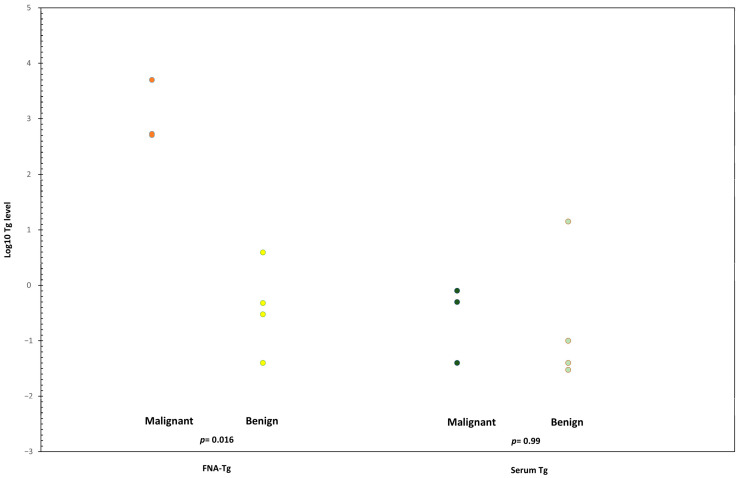
Tg values of the serum thyroglobulin antibody-positive patients. LN: lymph node; Tg: thyroglobulin.

A total of nine cases were determined to be positive for TgAb. Among the four TgAb-positive malignant cases, FNA-Tg levels were reported as 535, 508, 5000, and 5021 ng/mL, and serum Tg levels were 0.04, 0.04, 0.5, and 0.8 ng/mL, respectively (Table 3). The log-transformed Tg values are shown in Figure 3. Compared to the minimal differences observed in serum Tg levels, the FNA-Tg values demonstrated a significant distinction between the benign and malignant groups.

We investigated the association between FNA-Tg levels and various parameters including serum TSH, Tg, and TgAb levels. There were no positive correlations with FNA-Tg (Tg, ρ = 0.219, *p* = 0.159; TSH, ρ = 0.233, *p* = 0.133; TgAb, ρ = 0.088, *p* = 0.573).

## 3. Discussion

Given the consistent rate of malignant LN metastases in PTC, relatively limited accuracy of sonographic features, and occasional unreliability of cytological findings from fine-needle aspiration, there is a pressing need for alternative methods to validate LN metastases. This issue is particularly significant in Asian populations owing to their higher recurrence rates. For example, a study from China reported that 17% of the patients experienced cervical LN metastasis [25]. In contrast, local studies in Taiwan have reported recurrence rates as high as 23% [24]. Numerous studies have investigated the efficacy of FNA-Tg in differentiating between benign and malignant LNs and have established appropriate cutoff values [16,17,18,19,20,21]. The 2015 ATA guidelines also state that an FNA-Tg level < 1 ng/mL is considered reassuring to exclude LN metastasis [23]. However, the exact cutoff value remains a topic worthy of further investigation. At the same time, several confounding factors also warrant further investigation.

In our study, both groups had lower proportions of male participants, which may be related to the higher prevalence of thyroid cancer in females [26]. Additionally, we observed no significant difference in the likelihood of developing malignant LN metastasis during the follow-up between patients initially diagnosed without LN metastasis (N0 stage) and those diagnosed with N1 stage metastasis. This conclusion differs from that of the current ATA disease recurrence risk factors, which may be due to the small sample size in our study, potentially introducing statistical inaccuracies. Moreover, extending the follow-up period may have resulted in statistically significant differences. However, this also suggests the importance of careful follow-up even in patients initially classified as N0.

Regarding ultrasound imaging, while the malignant group demonstrated higher proportions of common malignant features than the benign group, only calcification exhibited a statistically significant difference, with only three cases identified. This highlights the fact that although ultrasound imaging aids in preliminary evaluations, it cannot provide definitive diagnostic accuracy. To improve diagnostic accuracy, additional techniques are needed. Recent studies have shown that advanced modalities such as microvascular imaging and contrast-enhanced ultrasound (CEUS) can enhance the detection of lymph node metastases in PTC [27,28]. Moreover, artificial intelligence (AI) models have demonstrated promising performance as adjunctive tools in ultrasound-based evaluation [29,30]. These emerging technologies warrant further investigation and may be considered for integration into future diagnostic protocols.

Apart from FNA-Tg and serum Tg, no significant differences were observed between the benign and malignant groups in other laboratory data or primary tumor conditions. FNA-Tg levels showed a significant difference between the two groups, with a median value of 5000 ng/mL in the malignant group versus 0.05 ng/mL in the benign group, which is consistent with findings from other studies conducted in Asia [17]. In the benign group, two lymph nodes exhibited unexpectedly high FNA-Tg levels—3162 ng/mL and 5000 ng/mL—which were comparable to the median value observed in the malignant group. A careful chart review revealed that both nodes had been classified as benign based solely on cytology results. As previously noted, cytology may fail to detect malignancy in certain cases. Therefore, these markedly elevated FNA-Tg values raised a strong suspicion of occult metastasis. After thorough discussion with the patients, RFA was performed for both lymph nodes. In one case, the patient’s serum Tg level decreased from 33 ng/mL to 0.3 ng/mL post-treatment. In the other case, the targeted lymph node showed complete regression during a 12-month follow-up period. These post-treatment responses further support the likelihood that both lymph nodes were indeed malignant.

In our investigation, a cutoff value of 4.23 ng/mL demonstrated excellent diagnostic performance, achieving a sensitivity of 100%, a specificity of 90.2%, and an overall accuracy of 93.6%. To further evaluate diagnostic efficacy, we compared this cutoff to other large-scale studies conducted in Asia, particularly in Taiwan [17,24]. When applying FNA-Tg > 1 ng/mL, the accuracy was 87.3%, whereas using FNA-Tg minus serum Tg > 1 ng/mL achieved an accuracy of 92%. Both are considered reliable diagnostic criteria. However, it is noteworthy that the sensitivity of FNA-Tg minus serum Tg levels > 1 ng/mL in our study was 95.5%, which may have resulted in missed diagnoses of certain malignant LNs. Conversely, FNA-Tg > 1 ng/mL, with its lower specificity, could lead to potential overdiagnosis.

A Korean study by Moon et al., which included both pre- and post-thyroidectomy patients, reported a cutoff value of 1 ng/mL [17]. In contrast, studies from China by Xiao et al. and Zhao et al. reported cutoff values ranging from 10 to 55 ng/mL [18,21,25]. The widely varying cutoff values reported have created uncertainty. These discrepancies may be attributed to differences in the study populations and ethnicities, criteria for final diagnosis, inclusion criteria (such as preoperative or postoperative status and different types of thyroid cancer), and variations in analytical methods. Several other factors should also be considered. First, differences in the measurement methods may have influenced the results. Variables such as the type of assay used by the laboratory, the volume of washout fluid during aspiration, and the occurrence of matrix effects can contribute to varying outcomes. Tg is a glycoprotein synthesized by follicular cells that is involved in the synthesis of thyroid hormones. Various assays are used to evaluate Tg levels, including the immunometric assay (IMA), radioimmunoassay (RIA), enzyme-linked immunosorbent assay (ELISA), and electrochemiluminescent assay (ECLIA). These assays differ in their functional sensitivities and the extent to which they are affected by TgAb [31,32]. Furthermore, the washout volumes are between 0.5 and 3.0 mL [19]. In our study, we used 1 mL of normal saline as the washout fluid. This finding is consistent with the findings of other studies. Borel et al. demonstrated that up to 1.0 mL of fluid is sufficient to collect more than 97% of Tg [33]. On the other hand, normal saline can potentially alter protein conformation, leading to a matrix effect that can increase the measured thyroglobulin levels by up to 25% [34,35]. This may explain the slight increase in FNA-Tg levels observed in benign cases.

Second, whether the thyroid gland is present at the time of FNA-Tg sampling and how serum Tg levels influence FNA-Tg levels are worthy of discussion. In our study, there was no positive correlation between serum Tg and FNA-Tg levels. However, we initially excluded untreated patients. In the malignant group, all patients underwent total thyroidectomy followed by radioactive iodine therapy. Regarding patients in the benign group, 11 cases had received RFA to preserve the thyroid gland. When comparing the FNA-Tg levels within the benign group (thyroid preserved vs. total thyroid removed), there were no significant differences (Table 4). Nevertheless, despite studies by Bournaud et al. and Kim et al., which reported an inability to identify any influence of the presence of the thyroid gland [36,37], several other studies have found that high serum Tg levels can affect FNA-Tg values. Studies by Baskin et al. [34] and Uruno et al. [38] demonstrated that using FNA-Tg to diagnose LNs while the thyroid is still present results in a higher false-positive rate. D’angeli et al. also found that the use of lower thresholds could result in false-positive results [39]. This situation is particularly likely to occur when the serum Tg levels are elevated. Given these conflicting results, the current study aimed to establish different diagnostic thresholds for patients with and without total thyroidectomy. For example, Zhao et al. [21] established cutoff values of 55.99 ng/mL for patients with intact thyroids and 9.71 ng/mL for those post-thyroidectomy, achieving high sensitivity (95.1%, 96.7%) and specificity (100%, 100%). Similar findings were observed in the study by Frasoldati et al. [11]. Unfortunately, in our study, owing to the small number of patients with thyroid preservation and the fact that all LNs were benign, we were unable to establish different diagnostic thresholds.

Third, there is a need to discuss TgAbs. Given that the same methods were used to measure FNA-Tg and serum Tg and that serum Tg is significantly affected by TgAb, we hypothesized that TgAb would also affect FNA-Tg. However, Boi et al. [40] found that the presence of Tg Abs does not affect the diagnostic accuracy of FNA-Tg. This can possibly be explained by the findings of Sigstad et al. [41], who found that in serum TgAb-positive patients, TgAb was not detected in the FNA washout fluid. It is important to note that using FNA-Tg measurements allows us to avoid missing malignant LN metastases in patients who are serum TgAb-positive.

In our study, we identified nine TgAb-positive cases, four of which were in the malignant group. These TgAb-positive metastatic LNs exhibited varying sizes and their echo patterns did not display typical malignant features. Moreover, serum Tg levels were often within the normal range. Therefore, relying solely on traditional follow-up methods can lead to misdiagnoses. Nonetheless, all malignant cases were accurately diagnosed using the cutoff value established in our study. A statistical comparison between the two groups revealed a significant difference in FNA-Tg values (*p* = 0.016), whereas no such difference was observed in serum Tg levels. It is also noteworthy that among the five LNs classified as benign, one displayed an FNA-Tg value of 3.93 ng/mL, which is close to our designated cutoff. Upon further investigation, we found that the patient did not undergo total thyroidectomy and had residual thyroid tissue. The elevated Tg level in this case could likely be attributed to the fine-needle aspiration passing through the residual thyroid tissue. This observation highlights the need to consider these factors when interpreting Tg values during diagnostic evaluation.

Finally, we discuss the effect of serum TSH levels on the FNA-Tg concentration. In our study, we did not observe a positive correlation between TSH and FNA-Tg levels. Additionally, rhTSH stimulation was not used during FNA-Tg sampling. Cappelli et al. [42] reported that FNA-Tg shifted from undetectable to detectable levels in LNs following rhTSH stimulation in two patients. However, owing to small sample sizes and methodological biases, this approach is currently not widely adopted. Subsequently, Zanella et al. [43] observed no significant difference in the median FNA-Tg values between two groups categorized by TSH levels (0.07 vs. 82.2 mIU/mL; FNA-Tg 3.3 vs. 3.8 ng/mL, *p* = 0.2). In a study by Moon et al. [17], FNA-Tg levels in the benign group remained unaffected; however, there was a positive correlation between FNA-Tg and TSH elevation in the malignant group. This suggests the need for further investigation and a longer follow-up period in future studies.

Our study aimed to establish a cutoff value for FNA-Tg levels for local physicians, but with some limitations. First, the small sample size undeniably affected the reliability of our results. Further validation using larger, multicenter cohorts will be necessary to confirm our findings and increase generalizability. Second, in patients with residual thyroid tissue, passing the FNA through the tissue can result in false-positive Tg levels. Finally, our cutoff points were primarily based on FNA cytology results. Because patients with malignant cytology results often proceed directly to RFA treatment, definitive pathology reports from surgery are lacking, which introduces a potential source of bias. However, current guidelines suggest RFA as an alternative to surgical neck dissection for recurrent LN metastasis in patients at high surgical risk or in those who decline surgery [44,45]. As the number of patients undergoing RFA for direct treatment of LN metastasis is expected to rise, the challenge lies in accurately diagnosing malignant LNs prior to surgical pathology reports, while making treatment decisions that do not compromise patient outcomes. This underscores the significant clinical value of FNA-Tg.

## 4. Materials and Methods

### 4.1. Patients

**Figure 4:**
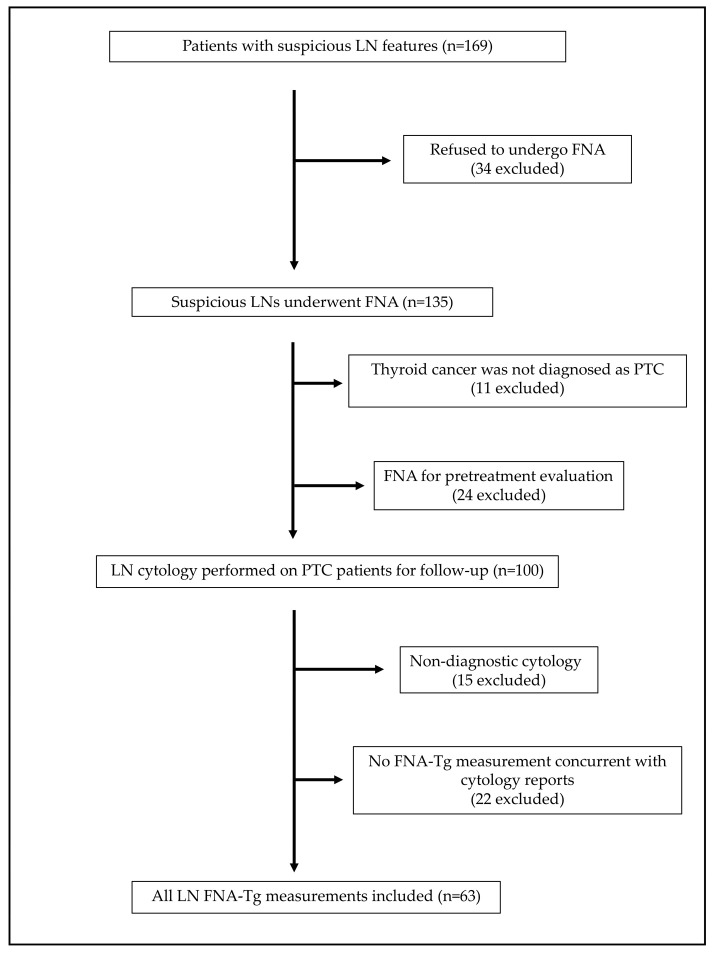
Flow diagram of patient enrollment. LN: lymph node, FNA: fine-needle aspiration, PTC: papillary thyroid carcinoma, FNA-Tg: thyroglobulin from fine-needle aspiration washout fluid.

We retrospectively identified 169 patients with suspicious LN features from the imaging department database of the Kaohsiung Chang Gung Memorial Hospital between January 2023 and June 2024. Patients who refused to undergo FNA were excluded, resulting in 135 LNs that underwent FNA. Further exclusion criteria included cases in which thyroid cancer was not diagnosed as PTC, FNA was performed solely for pretreatment evaluation, cytology was non-diagnostic, or cytology was performed without concurrent FNA-Tg measurement. Ultimately, 63 LNs from 60 patients were included in this study (Figure 4). Baseline characteristics including sex, age, and serum Tg, TSH, and TgAb levels were analyzed. Most importantly, FNA-Tg levels were compared with the LN cytology or pathology results. This study was approved by the Institutional Review Board of the Chang Gung Medical Foundation (zip code: 202401692B0).

### 4.2. Measurement Methods and Biochemical Analysis

**Figure 5:**
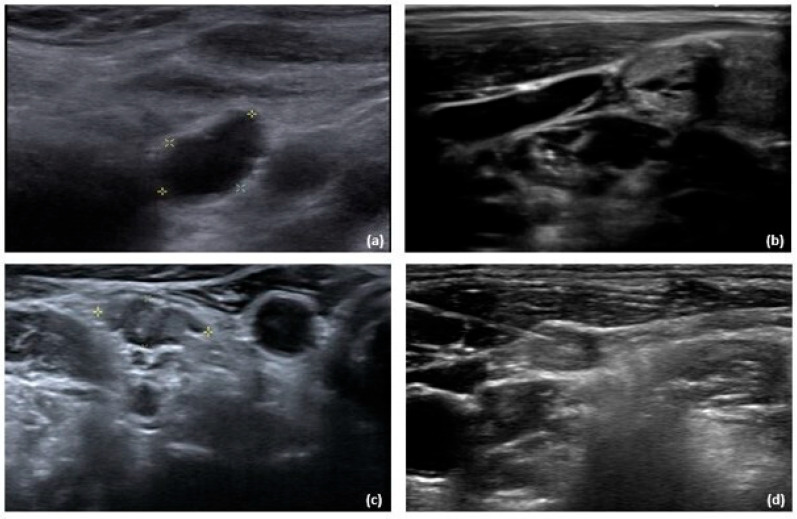
Cervical lymph nodes exhibiting malignant features and the fine-needle aspiration procedure. (**a**) Cystic content. (**b**) Calcification. (**c**) Hyperechoic content. (**d**) Fine-needle aspiration procedure.

Textures of LNs were evaluated via Siemens S2000™ ultrasound (Siemens medical solutions, Malvern, PA, USA). Cervical LNs detected on ultrasound that are >5 mm in the shortest diameter and/or exhibit suspicious malignant features, such as calcification, cystic change, absence of hilum, or hyperechogenicity [23,24], may be subjected to fine-needle aspiration evaluation (Figure 5). Under ultrasound guidance, two separate fine-needle aspirations were performed with a 23-gauge needle, one for cytological evaluation and the other for Tg washout analysis. Cytology specimens were initially fixed with Papanicolaou stain and reviewed by a specialized pathologist. Cytological results were presented using the Bethesda System [46] for reporting thyroid cytopathology.

The FNA-Tg specimens were washed with 1 mL of normal saline and analyzed by an electrochemiluminescent assay (ECLIA) on an Elecsys Cobas automated analyzer (ROCHE, Basel, Switzerland). The detection range was from <0.04 ng/mL to >5000 ng/mL. The threshold values corresponding to the 2.5th and 97.5th percentiles were 3.5–77 ng/mL. TgAb levels were assessed using the same ECLIA with a detection range of 10–4000 IU/mL. A positive result was defined as a value > 115 IU/mL. TSH levels were measured using a chemiluminescent microparticle immunoassay (CMIA) (TSH Reagent kit, Alinity I, Abbott, Chicago, IL, USA), which had an analytical measuring interval of 0.0083–100 mIU/L. The serum levels of Tg, TgAb, and TSH were measured concurrently during the FNA procedure, with a time difference of no more than a week.

### 4.3. Statistical Analysis

All statistical analyses were conducted using SPSS 25.0 statistical software for Windows (SPSS Inc., Chicago, IL, USA). Statistical significance was set at a *p* value < 0.05. Values with a normal distribution were presented as mean ± SD, while values with a non-normal distribution were presented as median (interquartile range). Normally distributed variables were compared between groups using the independent samples t-test, whereas variables with a non-normal distribution were compared using the Mann–Whitney U test. Categorical variables were compared using the chi-square and Fisher’s exact tests. Additionally, receiver operating characteristic (ROC) curve analysis was conducted to determine the cutoff value of FNA-Tg for diagnosing LN metastasis. The area under the ROC curve (AUC) and confidence intervals (CIs) were evaluated. Spearman’s rank correlation was used to assess the relationships between FNA-Tg and other parameters.

## 5. Conclusions

Our results validated 4.23 ng/mL of FNA-Tg as the cutoff value for diagnosing LN metastasis in PTC. The use of FNA-Tg may be beneficial for the diagnosis of local LN recurrence in PTC patients who have undergone total thyroidectomy or RFA. Furthermore, FNA-Tg may provide valuable diagnostic insights for patients with residual thyroid tissue or positive thyroglobulin antibodies. In our analysis, potential confounding factors such as serum Tg and TgAb levels did not significantly affect FNA-Tg performance. We anticipate that future large-scale studies will significantly contribute to the analysis of specific values and outcomes.

## Figures and Tables

**Table 1:** The baseline characteristics of all patients, as well as those categorized based on the final diagnosis of LNs.

	All Patients (n = 63)	Final Diagnosis		
		Malignancy (n = 22)	Benign (n = 41)	*p* Value
**Patients’ characteristics**				
Sex (males/females)	[15/48]	[4/18]	[11/30]	0.442
Age, y	50 ± 13.1	50.5 ± 14.9	50 ± 12.1	0.990
**Primary tumor**				
Size, mm	15 (18)	18 (14.2)	16 (19)	0.266
LN metastasis, % [yes/no]	41.2% [26/37]	54.5% [12/10]	34.1% [14/27]	0.179
Distant metastasis, % [yes/no]	3.2% [2/61]	9% [2/20]	0% [0/41]	0.118
**Follow-up LNs**				
Size, mm	8 (4)	7.5 (3.7)	8 (4)	0.873
Calcification (yes/no)	[3/60]	[3/19]	[0/41]	0.039
Cystic content (yes/no)	[7/56]	[4/18]	[3/38]	0.281
Hyperechoic content (yes/no)	[14/49]	[6/16]	[8/33]	0.534
Absence of hilum (yes/no)	[38/25]	[17/5]	[21/20]	0.060
**Lab data**				
Serum Tg, ng/mL	1.67 (12.7)	4.8 (20.4)	0.7 (10.8)	0.021
Serum Tg antibody (yes/no)	[9/54]	[4/18]	[5/36]	0.133
Serum TSH, µIU/mL	0.4 (1.9)	0.24 (1.47)	0.50 (2.04)	0.065
FNA-Tg, ng/mL	0.4 (976)	5000 (4447)	0.04 (0.26)	<0.001

Data are expressed as mean ± SD or median (interquartile range). LN: lymph node; Tg: thyroglobulin; TSH: Thyroid-Stimulating Hormone, FNA-Tg: thyroglobulin from fine-needle aspiration washout fluid.

**Table 2:** Comparison of diagnostic accuracies across different FNA-Tg cutoff values.

	Sensitivity	Specificity	PPV	NPV	Accuracy
FNA-Tg > 4.23 ng/mL	100%	90.2%	84.6%	100%	93.6%
FNA-Tg > 1 ng/mL [17,23]	100%	80.5%	73.3%	100%	87.3%
FNA-Tg-Serum Tg > 1 ng/mL [24]	95.5%	90.2%	84.4%	97.4%	92%

Tg: thyroglobulin; FNA-Tg: thyroglobulin from fine-needle aspiration washout fluid.

**Table 3:** Analysis of serum thyroglobulin antibody-positive cases.

Serum Tg Antibody (+)	FNA-Tg, ng/mL	Serum Tg, ng/mL	LN Size, mm
Malignancy-Case 1	535	0.8	8
Malignancy-Case 2	5000	0.5	13
Malignancy-Case 3	5021	0.04	6
Malignancy-Case 4	508	0.04	5
Benign-Case 1	0.04	0.1	8
Benign-Case 2	0.3	0.1	7
Benign-Case 3	0.48	0.03	5
Benign-Case 4	0.04	0.04	11
Benign-Case 5	3.93	14.2	8

LN: lymph node; Tg: thyroglobulin.

**Table 4:** FNA-Tg and serum Tg levels within the benign group.

Benign Group	Thyroid Preserved	Total Thyroid Removed	*p* Value
Cases, n	11	30	
FNA-Tg, ng/mL	0.05 (0.18)	0.04 (0.14)	0.607
Serum Tg, ng/mL	9.22 (19.2)	0.2 (5.57)	0.027

Data are expressed as median (interquartile range). FNA-Tg: thyroglobulin from fine-needle aspiration washout fluid; Tg: thyroglobulin.

## Data Availability

The data of the current study are available from the corresponding author upon reasonable request.
